# Clinical audit system in implementing Surviving Sepsis Campaign guidelines in patients with peritonitis

**DOI:** 10.1186/cc14033

**Published:** 2014-12-03

**Authors:** RC Valiveru, NK Maroju, K Srinivasan, A Cherian

**Affiliations:** 1 Department of Surgery, Jawaharlal Institute of Postgraduate Medical Education and Research, Dhanvantari Nagar, Pondicherry, India; 2 Department of Anesthesiology and Critical Care, Jawaharlal Institute of Postgraduate Medical Education and Research, Dhanvantari Nagar, Pondicherry, India

## Introduction

Sepsis is the predominant cause of morbidity and mortality in patients with peritonitis [1-6]. The Surviving Sepsis Campaign (SSC) is an international effort in reducing mortality based on evidence-based guidelines [7-13]. This study aims to assess the impact of audit-based feedback in a Plan-Do-Study-Act (PDSA) format on improving implementation of the SSC guidelines in patients with generalised peritonitis at our centre.

## Methods

This prospective observational study was conducted in four audit cycles in PDSA format. Multidepartmental inputs were taken to suggest modifications in practice. A questionnaire-based analysis of reasons for noncompliance was done to find out the opinions and reasons for noncompliance. Morbidity, mortality, the ICU and hospital stay among these patients were also analysed.

## Results

**Figure 1:**
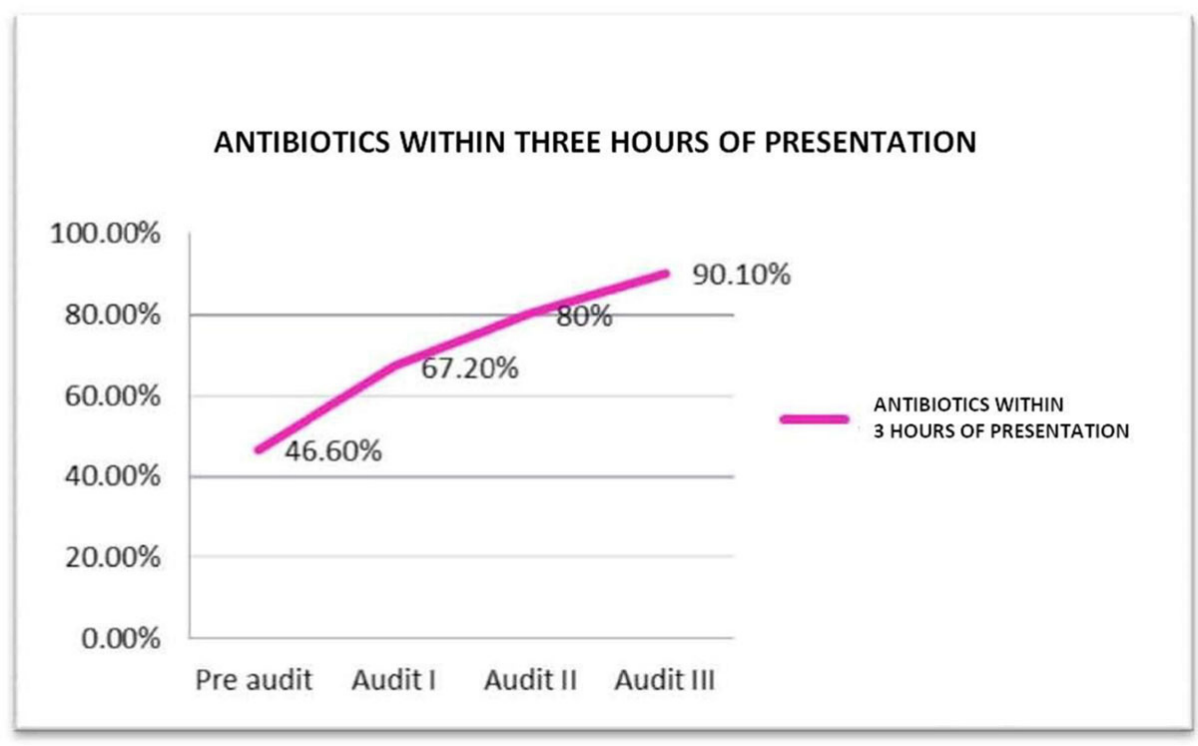
**Compliance with antibiotic administration within 3 hours of presentation**.

**Figure 2:**
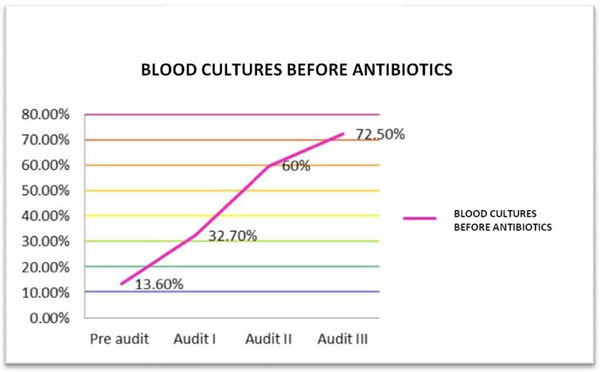
**Compliance with obtaining blood cultures before antibiotic administration**.

**Figure 3:**
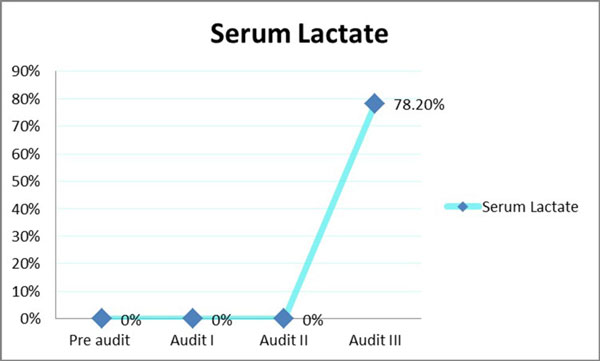
**Compliance with measurement of serum lactate**.

**Figure 4:**
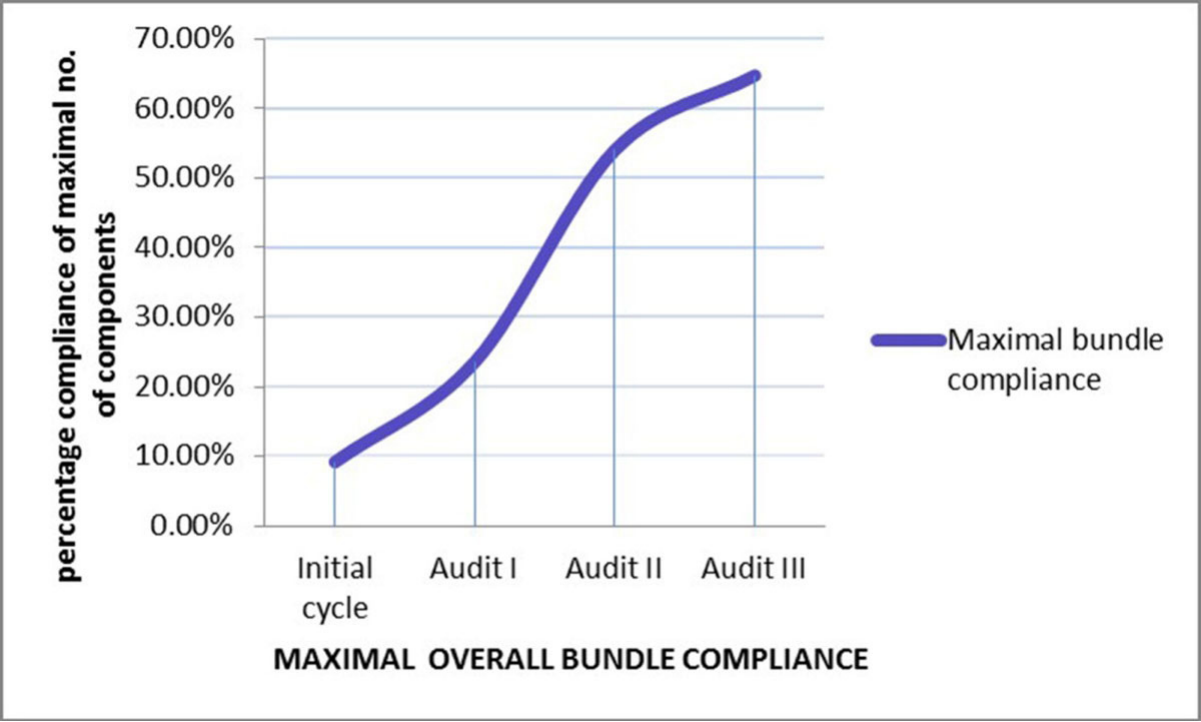
**Overall bundle compliance**.

**Figure 5:**
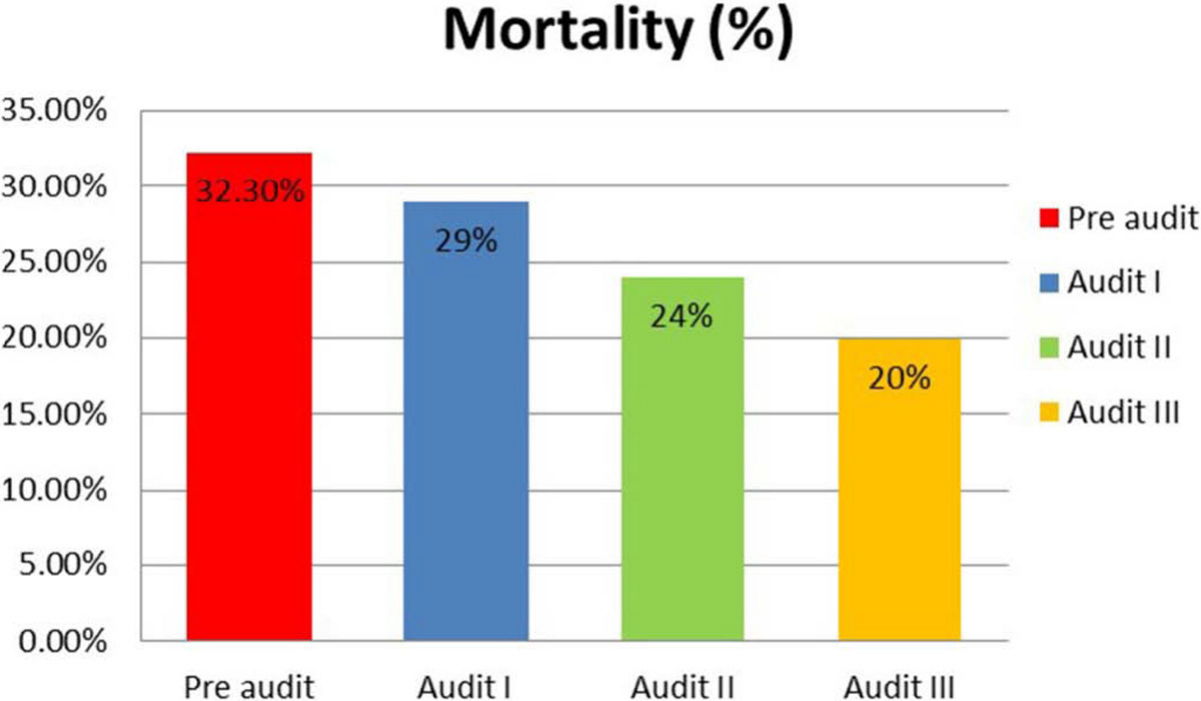


The baseline compliance with i.v. bolus administration, CVP-guided fluids and inotrope supports when indicated were 100%. Over the course of the three audit cycles, statistically significant improvement in compliance was noted for antibiotic administration within 3 hours of presentation (46% to 90%) (Table 1 Figure 1), obtaining blood cultures before antibiotics (13.8% to 72.5%) (Table 1 Figure 2) and serum lactate measurement (0% to 78.2%) (Figure 3). Overall bundle compliance improved from 9.2% to 64.7% (Table 2 Figure 4) by the end of Audit III. The mortality rate decreased from 32.3% to 20% (Table 2 Figure 5).

**Table 1:** Compliance with obtaining blood cultures before antibiotics and antibiotic administration within 3 hours.

Number of patients	Pre audit (*n *= 65)	Audit I (*n *= 55)	Audit II (*n *= 50)	Audit III (*n *= 51)
Blood cultures obtained before antibiotics	9 (13.6%)	18 (32.7%)^a^	30 (60%)^b^	37 (72.54%)^b^
Antibiotics given within 3 hours	30 (46.1%)	30 (67.2%)^c^	40 (80%)^d^	46 (90.1%)^b^

^a^*P *= 0.016. ^b^*P *< 0.0001. ^c^*P *= 0.463. ^d^*P *= 0.0002.

**Table 2:** Total bundle compliance.

Total number of bundle components performed	Pre audit (*n *= 65)	Audit I (*n *= 55)	Audit II (*n *= 50)	Audit III (*n *= 51)
**6**	0	0	0	33^a ^(64.7)
**5**	6 (9.2%)	13 (23.6%)	27 (54%)	3 (5.8%)
**4**	26 (40%)	29 (52.7%)	16 (32%)	11 (21.5%)
**3**	33 (50.7%)	13 (23.6%)	7 (14%)	4 (7.8%)
**2**	0	0	0	0
**1**	0	0	0	0

^a^Serum lactate was available in the hospital only during audit cycle III.

## Conclusion

This study demonstrates that audit-based feedback is a dependable means of improving compliance with SSC guidelines. It brings about improvement by educating users, by modifying their behaviour through feedback and also enhances process improvement by identifying and correcting systemic deficiencies in the organisation.

**Table 3:** Overall mortality.

	Pre audit (*n *= 65)	Audit I (*n *= 55)	Audit II (*n *= 50)	Audit III (*n *= 51)
Overall mortality in percentage	21 (32.3%)	16 (29%)	12 (24%)	11 (20%)
*P *value (compared with the initial cycle)		0.843	0.407	0.143

## References

[B1] Afridi SP Malik F Ur-Rahman S Shamim S Samo KA Spectrum of perforation peritonitis in Pakistan: 300 cases Eastern experience World J Emerg Surg 2008 3 31 10.1186/1749-7922-3-31 18992164 PMC2614978

[B2] Chakma SM Singh RL Parmekar MV Singh KHG Kapa B Sharatchandra KH Longkumer AT Rudrappa S Spectrum of perforation peritonitis J Clin Diagn Res 2013 7 2518 2520 24392388 10.7860/JCDR/2013/5768.3596 PMC3879863

[B3] Jimenez MF Marshall JC International Sepsis Forum: source control in the management of sepsis Intensive Care Med 2001 27 Suppl 1 S49 S62 11307370 10.1007/pl00003797

[B4] Bali RS Verma S Agarwal PN Singh R Talwar N Perforation peritonitis and the developing world ISRN Surg 2014 2014 105492 25006512 10.1155/2014/105492 PMC4004134

[B5] Søreide K Thorsen K Søreide JA Strategies to improve the outcome of emergency surgery for perforated peptic ulcer Br J Surg 2014 101 e51 e64 10.1002/bjs.9368 24338777

[B6] Noguiera C Silva AS Santos JN Silva AG Ferreira J Matos E Vilaça H Perforated peptic ulcer: main factors of morbidity and mortality World J Surg 2003 27 782 787 10.1007/s00268-003-6645-0 14509505

[B7] Dellinger RP Levy MM Rhodes A Annane D Gerlach H Opal SM Sevransky JE Sprung CL Douglas IS Jaeschke R Osborn TM Nunnally ME Townsend SR Reinhart K Kleinpell RM Angus DC Deutschman CS Machado FR Rubenfeld GD Webb S Beale RJ Vincent JL Moreno R Surviving sepsis Campaign Guidelines Committee including The Pediatric Subgroup Surviving Sepsis Campaign: international guidelines for management of severe sepsis and septic shock, 2012 Intensive Care Med 2013 39 165 228 10.1007/s00134-012-2769-8 23361625 PMC7095153

[B8] Marshall JC Dellinger RP Levy M The Surviving Sepsis Campaign: a history and a perspective Surg Infect 2010 11 275 281 10.1089/sur.2010.024 20524900

[B9] Castellanos-Ortega A Suberviola B García-Astudillo LA Holanda MS Ortiz F Llorca J Delgado-Rodríguez M Impact of the Surviving Sepsis Campaign protocols on hospital length of stay and mortality in septic shock patients: results of a three-year follow-up quasi-experimental study Crit Care Med 2010 38 1036 1043 10.1097/CCM.0b013e3181d455b6 20154597

[B10] Zambon M Ceola M Almeida-de-Castro R Gullo A Vincent JL Implementation of the Surviving Sepsis Campaign guidelines for severe sepsis and septic shock: we could go faster J Crit Care 2008 23 455 460 10.1016/j.jcrc.2007.08.003 19056006

[B11] Kang MJ Shin TG Jo IJ Jeon K Suh GY Sim MS Lim SY Song KJ Jeong YK Factors influencing compliance with early resuscitation bundle in the management of severe sepsis and septic shock Shock 2012 38 474 479 10.1097/SHK.0b013e31826eea2b 23042195

[B12] Wang Z Xiong Y Schorr C Dellinger RP Impact of sepsis bundle strategy on outcomes of patients suffering from severe sepsis and septic shock in china J Emerg Med 2013 44 735 741 10.1016/j.jemermed.2012.07.084 23332802

[B13] Li Z-Q Xi X-M Luo X Li J Jiang L Implementing surviving sepsis campaign bundles in China: a prospective cohort study Chin Med J (Engl) 2013 126 1819 1825 23673093

